# Endurance- and Resistance-Trained Men Exhibit Lower Cardiovascular Responses to Psychosocial Stress Than Untrained Men

**DOI:** 10.3389/fpsyg.2018.00852

**Published:** 2018-06-01

**Authors:** Peter Gröpel, Maren Urner, Jens C. Pruessner, Markus Quirin

**Affiliations:** ^1^Department of Applied Psychology: Work, Education and Economy, University of Vienna, Vienna, Austria; ^2^Institute of Psychology, Osnabrück University, Osnabrück, Germany; ^3^Department of Psychology, University of Konstanz, Konstanz, Germany

**Keywords:** physical activity, exercise, sport, stress, TSST, cortisol, heart rate

## Abstract

Evidence shows that regular physical exercise reduces physiological reactivity to psychosocial stress. However, previous research mainly focused on the effect of endurance exercise, with only a few studies looking at the effect of resistance exercise. The current study tested whether individuals who regularly participate in either endurance or resistance training differ from untrained individuals in adrenal and cardiovascular reactivity to psychosocial stress. Twelve endurance-trained men, 10 resistance-trained men, and 12 healthy but untrained men were exposed to a standardized psychosocial stressor, the Trier Social Stress Test. Measurements of heart rate, free salivary cortisol levels, and mood were obtained throughout the test and compared among the three groups. Overall, both endurance- and resistance-trained men had lower heart rate levels than untrained men, indicating higher cardiac performance of the trained groups. Trained men also exhibited lower heart rate responses to psychosocial stress compared with untrained men. There were no significant group differences in either cortisol responses or mood responses to the stressor. The heart rate results are consistent with previous studies indicating reduced cardiovascular reactivity to psychosocial stress in trained individuals. These findings suggest that long-term endurance and resistance trainings may be related to the same cardiovascular benefits, without exhibiting strong effects on the cortisol reactivity to stress.

## Introduction

The World Health Organization calls stress as one of the greatest health risks of the 21st century (Leka et al., 2003). Regular physical activity has been identified as one means of effective prevention, with convincing evidence of its protective effects against stress-related diseases (Gerber and Pühse, 2009; Goldstein, 2010; Li and Siegrist, 2012; Gerber et al., 2014). These benefits are mediated through a number of mechanisms, including reduced reactivity of the sympathetic nervous system and the hypothalamic-pituitary-adrenal (HPA) axis to psychological stressors (Hamer et al., 2006; Sothmann, 2006). Specifically, physically active individuals typically show lower cortisol increase (Rimmele et al., 2007, 2009; Klaperski et al., 2013, 2014), lower cardiovascular reactivity (Crews and Landers, 1987; Spalding et al., 2004; Forcier et al., 2006; Rimmele et al., 2007, 2009; Klaperski et al., 2013, 2014), and more rapid cardiovascular recovery (Jackson and Dishman, 2006) to laboratory stressors when compared to their less active counterparts.

The majority of studies that showed effects of physical activity on adrenal and cardiovascular reactivity to psychological stressors included aerobic exercises (endurance training) such as jogging, biking, and long distance running, with only a few investigations looking at effects of anaerobic exercises (resistance training) such as weight lifting (cf. Klaperski et al., 2012). Of the few studies that directly compared the two, Spalding et al. (2004) found that individuals who participated in either endurance or resistance exercise training for 6 weeks showed lower cardiovascular responses to a cognitive stressor than untrained controls, with endurance training yielding somewhat stronger effects. Blumenthal et al. (1988, 1990) compared type-A individuals who had completed a 12-week endurance program with type-A individuals who had completed a 12-week resistance training program. The authors observed greater reductions in cardiovascular stress reactivity in the participants who completed the endurance program. Although these studies appear to favor endurance training over resistance training in reducing stress responsiveness, the training time period might have been too short for resistance training to demonstrate stronger effects. Thus, evidence whether or not long-term resistance training has the same psychophysiological benefits as long-term endurance training is still missing from the literature.

The aim of this study was thus to test the effect of both long-term endurance and resistance trainings on psychosocial stress reactivity by including three groups differing in the type of training (endurance-trained men, resistance-trained men, and untrained controls). Several authors state that regular exercise leads to physiological adaptations which contribute to a reduced physiological reaction to stressors in general (Spalding et al., 2004; Hamer et al., 2006; Huang et al., 2013; Klaperski et al., 2013, 2014). *Endurance training* is known to enhance aerobic capacity and to induce adaptations that increase ventricular filling and decrease myocardial work, which in turn improve cardiac performance (i.e., enhanced stroke volume) (Spalding et al., 2004). This enables submaximal workloads to be negotiated with greater efficiency (e.g., at a lower heart rate and blood pressure; McArdle et al., 1996), resulting in less strain on the cardiovascular system. Notably, these improvements may generalize from ergogenic to psychogenic challenges (Sinyor et al., 1983; Claytor, 1991). Similar to endurance training, *resistance training* produces adaptations in the cardiovascular system that lower blood pressure (Spalding et al., 2004) and cause a more rapid return of heart rate to baseline levels following physical exercise (Darr et al., 1988), even though aerobic capacity does not increase as much (Spalding et al., 2004). In addition, in trained weightlifters, Bush et al. (1999) documented a significant increase in plasma peptide f (P-F) up to at least 240 min following exercise, which signals immune response leading to recovery from stress. Hence, although recent research related to physical activity and stress responsiveness mainly focused on endurance training, resistance training is likely to produce similar positive outcomes (see Huang et al., 2013, for profound discussion).

Based on the above evidence, we hypothesized that trained men, both endurance and resistance, would show lower endocrine and cardiovascular stress responses than untrained men. Endurance-trained men engaged in regular training in long distance running or biking for at least 1 year, whereas resistance-trained men participated in regular training in weight lifting for at least 1 year as well. Untrained men did not exercise regularly and their physical activity was limited to casual use of the bicycle as means of transportation. Psychosocial stress was induced by the Trier Social Stress Test (TSST; Kirschbaum et al., 1993) consisting of public speaking and a mental arithmetic task. Physiological responses to the stressor were assessed by repeated measurement of heart rate and free salivary cortisol levels. The level of baseline cortisol regulation was controlled for through the measurement of the cortisol awaking response (CAR).

In addition to physiological responses, we also measured psychological responses to stress, such as positive and negative mood. Even though psychosocial stress typically worsens mood (Kuhl, 2001), Rimmele et al. (2007) reported larger mood worsening in untrained men compared to trained men. Regular physical activity has been associated with higher self-efficacy (Netz et al., 2005) and self-efficacy has been associated with lower anxiety and physiological stress reactivity (Bandura, 1997). Therefore, we hypothesized that endurance and resistance trained men would show reduced mood worsening than untrained men in response to the TSST.

Because stress responsiveness may at least in part depend on personality traits (Kuhl, 2001), we controlled for action orientation which refers to the individual ability to efficiently cope with stress (Kuhl, 1994b). Individuals high in action orientation are characterized by superior coping skills, including the regulation of thoughts and emotions in favor of goal-related actions, whereas individuals low in action orientation are less resistant and prone to stick to adverse situations. In comparison to student controls, athletes typically have higher levels of action orientation (Beckmann and Kazén, 1994). Moreover, we had previously shown that individuals high in action orientation depict less increase in cortisol after a social stressor than those low in action orientation (Quirin et al., 2011). Therefore, we hypothesized that endurance and resistance trained men would score higher in action orientation than untrained men, and that the level of action orientation would correlate with physiological and psychological responses to the TSST.

## Materials and Methods

### Participants

An a priori sample-size calculation (G^∗^Power version 3.1.9.2; Faul et al., 2009) for three groups and two (or more) repeated measurements, based on middle-to-large effect size (*f* = 0.30), power = 0.80, and α = 0.05, resulted in a minimal sample size of 30 participants (i.e., 10 pro group). The anticipation of a middle-to-large effect size was based on previous research investigating the effect of physical activity on stress responsiveness (Klaperski et al., 2013, 2014).

The study sample comprised 12 endurance-trained men, 10 resistance-trained men, and 12 untrained men. All trained participants were recruited by local sport clubs, fitness studios, and through advertisements on regional web pages of endurance sports and at the Osnabrück University, Germany. In a telephone interview, we screened all participants for their weekly training schedule (duration, intensity, and frequency) and their onset of sportive activity in general. To be included, endurance-trained men needed to be engaged in intensive training (at least five scheduled units per week with at least one unit at a minimum of 80% of the maximal efficiency) for at least 1 year. Resistance-trained men needed to train intensively (at least four scheduled units per week with at least one unit resulting in complete muscle fatigue) for at least 1 year, as well. Untrained men were recruited through flyers at the Osnabrück University. They had to not exercise regularly to be included, and their physical activity had to be limited to casual use of the bicycle as means of transportation. General exclusion criteria were medication intake, reported medical illnesses (diabetes, cardiovascular diseases, and liver diseases), psychological treatment, head trauma, substance abuse, and smoking. All participants were native German speakers. This study was carried out in accordance with the recommendations of American Psychological Association with written informed consent from all subjects. Before entering the study, all participants gave written informed consent in accordance with the Declaration of Helsinki. The protocol was approved by the ethics committee of the Osnabrück University (#4/71040/0/6). After the experiment, participants were paid €25.

### Procedure

The experimental session lasted for 2 h and took place between 13:00 and 16:00 h in order to control for diurnal variations in cortisol levels (Dickerson and Kemeny, 2004). Participants were asked to refrain from eating, drinking (except water), and intensive physical activity for at least 1 h prior to the experiment. Upon arrival at the laboratory, participants were connected to a portable biosignal recorder for the electrocardiogram recording (Varioport; Becker-Meditec Inc., Karlsruhe, Germany). Following a baseline period of 30 min, in which participants filled out questionnaires, participants were introduced to the upcoming TSST task. The TSST is a standardized performance task protocol which reliably and validly elicits large and robust HPA responses (Dickerson and Kemeny, 2004). In this protocol, participants are exposed to a 5-min public speaking task (mock job interview) and a subsequent mental arithmetic task (serial subtraction) performed out loud in front of a video camera and two judges who keep a neutral expression throughout the task. Participants were given 5 min to prepare for the mock-job interview in which they were supposed to convince the two judges that they were the most suitable candidate for a position of their choice. They were also told that they would be videotaped for non-verbal behavioral analysis to be conducted later.

After the preparation period, participants were guided to the TSST room, where they stood in an upright standing position in front of a camera and two experimenters in white coats representing the judges. Participants were first asked to deliver the job speech. If a subject stopped early, some seconds of silence were kept and then a standardized catalog of general questions was used in order to keep the subject talking (e.g., “Which leadership qualities do you think you have?”). Following the speech, participants did the serial subtraction task: Starting at the value 2010, they were asked to count down to zero by serially subtracting the number 13 as quickly and accurately as possible. Every time a mistake occurred, they had to start over from 2010. After the 10-min stress protocol, participants were guided back to the first room, where they were instructed to sit quietly for 60 more minutes until saliva sampling was completed and to fill in some additional questionnaires. Finally, they were debriefed, received their compensation, and were dismissed.

In addition to the experimental session, participants collected saliva samples on the last 3 days before the test day. They were instructed to take three saliva samples on the three consecutive mornings prior to the test day immediately at awakening, 30 min, and 60 min thereafter. Participants were also instructed not to eat, to brush their teeth, and were only allowed to drink water before completing saliva sampling to avoid contamination of the sampling, and to refrain from sports in the 1st hour after awakening. No other instructions were given that could interfere with the participants’ normal daily routines. The majority of subjects typically show a marked cortisol increase of 50–80% within the first 30 min after awakening; a pattern known as “cortisol awakening response” (CAR; Schmidt-Reinwald et al., 1999). Compliance with the saliva collection protocol was electronically monitored (MEMS 6 TrackCap, AARDEX ltd., Zug, Switzerland). Of the total of 306 samples, 12 samples (3.9%) were excluded because of non-compliance with the protocol (e.g., large time period between two samples). Hence, in general, participants were conscientious about meeting their commitment.

### Endocrine Measures

Salivary free cortisol is a reliable and valid measure of the biologically active fraction of cortisol (Vining et al., 1983; Kirschbaum and Hellhammer, 1994). Salivary free cortisol gradually increases within about 10 min, and peaks around 10–30 min after stressor cessation (Foley and Kirschbaum, 2010). Saliva was collected using Salivette sampling devices (Sarstedt, Rommelsdorf, Germany). Participants were asked to keep the samples collected on the three morning prior to the test day in their freezers and return them to the laboratory on their test day. On that day, before and after the TSST, eight saliva samples were collected from each participant. The baseline was calculated by the mean of the first three samplings before the onset of the TSST (-20, -10, -2 min), the endocrine response to the psychosocial stressor was computed from the samples obtained after the onset of the TSST (+15, +30, +45, +60, +75 min), resulting in six repeated cortisol measures. Saliva samples were stored at -20°C and sent to the University of Trier (Germany) for biochemical analysis of free cortisol concentration. Cortisol was analyzed by a time-resolved immunoassay with fluorescence detection (Dressendörfer et al., 1992). Intra- and interassay coefficients of variation were below 9.0%.

### Autonomic Measures

Heart rate was monitored for subsequent 60-s segments using a portable biosignal recorder (Varioport; Becker-Meditec Inc., Karlsruhe, Germany). For the baseline, four segments measured 10 min after the subject started to answer the questionnaires were averaged. For the stress phase, nine consecutive 60-s segments after the onset of the TSST were used. For the recovery phase, the first 4 min after the TSST were averaged.

### Psychological Measures

Action orientation was assessed with the two-dimensional Action Control Scale (ACS-90; Kuhl, 1994a): the Demand-Related Action Control subscale addresses how people cope with upcoming difficulties and demands, and the Threat-Related Action Control subscale focuses on coping with experienced failures and threats. Perceived life stress was measured with the one-dimensional Perceived Stress Scale (PSS; Cohen and Williamson, 1988) that addresses unpredictability, uncontrollability, overload, and general levels of experienced stress during the last month. At the baseline and after the TSST, positive and negative mood was repeatedly measured with the Positive and Negative Affect Schedule (PANAS; Watson et al., 1988). All questionnaires have been broadly used and have shown satisfactory reliability and validity. The calculated Cronbach’s alphas show good internal consistency for the demand-related and the threat-related subscales of the ACS-90 (α = 0.86 and 0.79, respectively), for the PSS (α = 0.81), and for the PANAS (all αs > 0.70).

### Statistical Analysis

Psychological and physiological data were analyzed using two-way repeated measure analysis of variance (ANOVA) with group as the between-subject factor (3 groups: endurance-trained men vs. resistance-trained men vs. untrained men) and time as the within-subject factor (repeated measures: 3 for the CAR, 6 for cortisol at the test day, 11 for heart rate, and 2 for positive and negative mood). Repeated-measures results were verified with Greenhouse-Geisser corrections where the Mauchly test of sphericity determined heterogeneity of covariance. Group differences were determined using separate one-way ANOVAs. *Post hoc* comparisons were performed with Bonferroni adjustment. We additionally calculated the “area under the individual response curve with respect to the increase” (AUC_I_) using the trapezoid formula (Pruessner et al., 2003), which allows a sensitive measurement of changes over time (Hellhammer et al., 2007). To test our prediction that trained men, both endurance and resistance, would show lower stress responses than untrained men, a contrast-coded test was conducted on AUC_I_ in the case that ANOVA revealed significant group differences. The contrast to test this prediction was -0.5, -0.5, +1, which indicated lower AUC_I_ for trained men than untrained men. All statistical analyses were performed using SPSS 24.0 (IBM Corp.; Armonk, NY, United States). Data was preprocessed with scatterplots to detect possible outliers; values that differed more than three standard deviations from the mean were excluded. Data are presented as mean ± SEM. The level of significance was set at *p* < 0.05 (two-tailed). Effect size was tested by the partial eta squared ($\eta_{p}^{2}$) which indicates the proportion of total variability attributable to a factor.

## Results

Demographic and psychological characteristics of the three groups are presented in **Table 1**. The groups did not significantly differ in size, weight, and BMI (all *p* > 0.15). A significant difference in age was found among the groups, *F*(2,31) = 5.91, *p* = 0.01, $\eta_{p}^{2}$ = 0.28. Resistance-trained men were significantly younger than endurance-trained men (*post hoc* analysis, *p* = 0.01). Untrained men did not differ in age from endurance-trained men and resistance-trained men. Because of these group differences in age, we additionally controlled for age in the following analyses.

**Table 1:** Demographic and psychological characteristics of the groups.

	Endurance (*n* = 12)	Resistance (*n* = 10)	Untrained (*n* = 12)
Age (years) ^∗∗^	25.92 @ 1.31	21.10 @ 0.91	23.92 @ 0.47
Size (meter)	1.81 @ 0.02	1.80 @ 0.03	1.81 @ 0.02
Weight (kg)	72.33 @ 2.37	78.30 @ 3.29	76.08 @ 3.17
Body mass index (kg/m^2^)	21.98 @ 0.41	23.99 @ 0.49	23.24 @ 1.01
Perceived stress (PSS)	21.67 @ 2.92	20.90 @ 2.70	24.17 @ 2.46
Demand-related action orientation (ACS-90)^∗∗^	8.42 @ 0.86	6.89 @ 1.27	3.33 @ 0.71
Threat-related action orientation (ACS-90)	7.75 @ 0.96	5.56 @ 0.97	5.17 @ 0.85

*Data are presented as mean ± SEM; ^∗∗^p < 0.01.*

### Action Orientation

The groups differed significantly in demand-related action orientation, *F*(2,30) = 8.65, *p* = 0.001, $\eta_{p}^{2}$ = 0.37, but not in threat-related action orientation, *F*(2,30) = 2.38, *p* = 0.11, $\eta_{p}^{2}$ = 0.14. For demand-related action orientation, the group of untrained men differed significantly from both the endurance group (*post hoc* analysis, *p* = 0.001) and the resistance group (*post hoc* analysis, *p* = 0.04), showing lower values. The two latter groups did not differ in the level of demand-related action orientation (*post hoc* analysis, *p* = 0.80). Although trained and untrained men differed in demand-related action orientation, the level of demand-related action orientation did not affect stress responses in the present sample; it did not correlate with either AUC_I_ CAR, or AUC_I_ cortisol, or AUC_I_ heart rate or differences in positive and negative mood from before to after the TSST (*p* > 0.26). Similarly, threat-related action orientation did not correlate with either CAR or physiological responses to stress (*p* > 0.39). There was only a marginal correlation between threat-related action orientation and differences in negative mood from before to after the TSST which became significant after controlling for age (*r*_partial_ = -0.37, *p* = 0.04), indicating that, regardless of group, participants higher in threat-related action orientation did not increase negative affect from before to after the TSST as much as those low in threat-related action orientation.

### CAR and Chronic Stress

**FIGURE 1:**
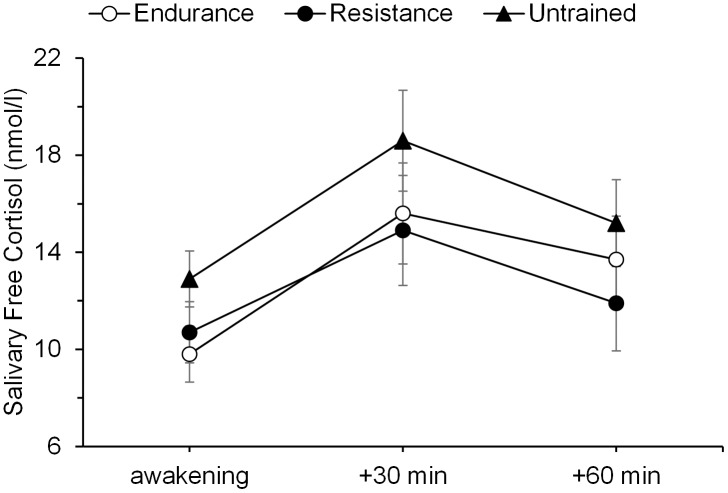
Cortisol awakening responses. Mean salivary free cortisol levels at awakening, 30 min, and 60 min thereafter in endurance-trained men, resistance-trained men, and untrained men. Error bars are standard errors of the mean (SEM).

To test whether the groups differed in the CAR or in chronic stress, three cortisol parameters were computed by averaging over 3 days: The mean cortisol level immediately after awakening, the mean cortisol level 30 min after awakening, and the mean cortisol level 60 min after awakening. The 3 days were not different from each other before averaging. The CARs are depicted in **Figure 1**. A repeated-measures ANOVA revealed a main effect of time, *F*(2,62) = 12.09, *p* = 0.001, $\eta_{p}^{2}$ = 0.28, indicating a significant increase in cortisol levels after awaking across all groups. The groups did not significantly differ in the CAR (main effect of group, *p* = 0.27; group by time interaction, *p* = 0.87). Similarly, the groups did not differ in the AUC_I_ CAR, *p* = 0.74. In addition, there were no difference in self-reported levels of perceived life stress among the three groups (*p* = 0.68; see **Table 1**). The inclusion of age as covariate did not significantly change the results. These results indicate that the study groups did neither differ in basic regulation of the HPA, nor in the level of chronic stress and thus any different responses to the TSST between the groups cannot be attributed to differences on these variables.

### Cortisol Responses to Stress

**FIGURE 2:**
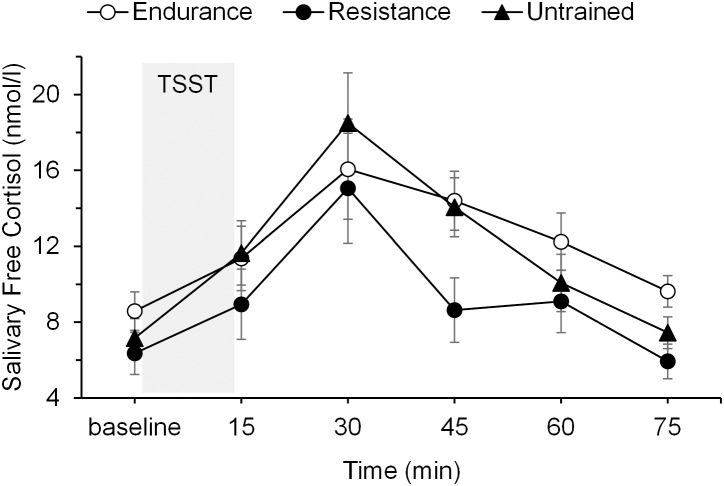
Mean salivary free cortisol levels before, during, and after the psychosocial stressor (TSST, shaded area) in endurance-trained men, resistance-trained men, and untrained men. Error bars are standard errors of the mean (SEM).

Cortisol values of one resistance-trained man differed more than three standard deviations from the mean and his data was therefore excluded from the analysis. The psychosocial stressor induced a significant increase in salivary free cortisol levels in all three groups, as illustrated by a significant main effect of time, *F*(2.72,81.55) = 28.09, *p* = 0.001, $\eta_{p}^{2}$ = 0.48. There were no significant differences in cortisol levels between the groups at baseline (*p* = 0.46). The group also did not differ in their cortisol responses to the stressor, as indicated by an non-significant main effect of group, *F*(2,30) = 2.65, *p* = 0.087, $\eta_{p}^{2}$ = 0.15, and non-significant group by time interaction, *F*(5.44,81.55) = 1.98, *p* = 0.084, $\eta_{p}^{2}$ = 0.12. Mean salivary cortisol levels are presented in **Figure 2**. For cortisol increase (baseline to +75 min), a one-way ANOVA with AUC_I_ revealed no significant differences between the groups (*p* = 0.10). The inclusion of age as a covariate revealed the main effect of age, *F*(1,29) = 5.57, *p* = 0.03, $\eta_{p}^{2}$ = 0.16. Older participants showed stronger cortisol responses to the stressor regardless of group. Controlling for age did not result in significant differences in cortisol responsiveness among the groups.

### Heart Rate Responses to Stress

**FIGURE 3:**
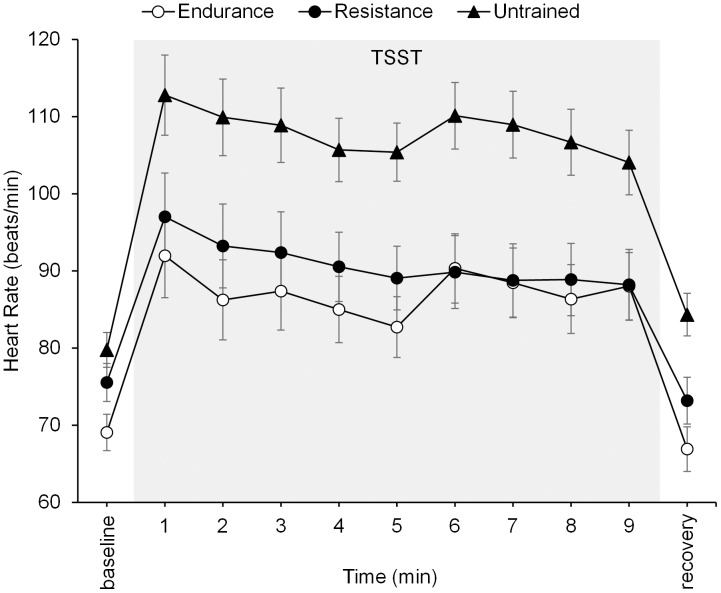
Mean heart rates before, during, and after the psychosocial stressor (TSST, shaded area) in endurance-trained men, resistance-trained men, and untrained men. Error bars are standard errors of the mean (SEM).

Heart rate at baseline differed significantly among groups, *F*(2,31) = 5.91, *p* = 0.01, $\eta_{p}^{2}$ = 0.28, with the lowest heart rate occurring in the group of endurance-trained men (69.04 ± 2.22 bpm), a medium heart rate in the group of resistance-trained men (75.54 ± 2.43 bpm), and the highest heart rate in the group of untrained men (79.76 ± 2.22 bpm). *Post hoc* tests revealed that the endurance group’s baseline differed significantly from that in the untrained group (*p* = 0.01), whereas the difference between the endurance and the resistance groups and the difference between the resistance and the untrained groups were not significant (*p* = 0.17 and *p* = 0.63, respectively). The psychosocial stressor induced a significant increase in heart rate in all three groups, as illustrated by a significant main effect of time, *F*(3.62,108.53) = 44.84, *p* = 0.001, $\eta_{p}^{2}$ = 0.60. Furthermore, the average level of heart rate differed between the three groups [main effect of group: *F*(2,30) = 7.62, *p* = 0.002, $\eta_{p}^{2}$ = 0.34], with untrained men having the highest heart rate levels (*post hoc* analysis, *p* = 0.003 and *p* = 0.02 as compared to endurance-trained men and resistance-trained men, respectively). The endurance and resistance groups did not differ significantly in heart rate levels (*post hoc* analysis, *p* = 1.00). The group by time interaction was not significant (*p* = 0.22). Mean heart rate levels are presented in **Figure 3**. For heart rate increase (baseline to recovery), a one-way ANOVA with AUC_I_ revealed significant differences between groups, *F*(2,30) = 3.29, *p* = 0.05, $\eta_{p}^{2}$ = 0.18. Contrast-coded analysis (-0.5, -0.5, +1 for endurance-trained, resistance-trained, and untrained men, respectively) was significant, *t*(30) = 2.53, *p* = 0.017, indicating that lower heart rate increase was observed for trained men than untrained men. However, the more conservative simple *post hoc* tests (Bonferroni adjusted) were not significant. Here, the untrained group had higher but non-significant heart rate increase than the endurance group (*post hoc* analysis, *p* = 0.20) and the resistance group (*post hoc* analysis, *p* = 0.07), whereas the two latter groups did not differ from each other (*post hoc* analysis, *p* = 1.00). The difference in statistical significance based on which *post hoc* test is used is in line with some researchers recommending the more conservative Bonferroni adjustment (e.g., Bland and Altman, 1995; Greenhalgh, 1997), while others argue against it because it favors the general null hypothesis and considers important differences between pairs non-significant (e.g., Perneger, 1998). The observed group differences remained stable after including age as covariate in the above analyses.

### Psychological Responses to Stress

Negative mood significantly increased from before to after the stress protocol in all groups [main effect of time: *F*(1,31) = 17.01, *p* = 0.001, $\eta_{p}^{2}$ = 0.36]. No significant differences were observed between groups. Specifically, both the main effect of group (*p* = 0.83) and the group by time interaction (*p* = 0.62) were non-significant. Positive mood did not significantly change from before to after the TSST (main effect of time: *p* = 0.10). The main effect of group (*p* = 0.11) and the group by time interaction (*p* = 0.86) were also non-significant. Neither controlling for age or for action orientation resulted in significant group differences in either positive or negative mood. Mean positive and negative affect levels are presented in **Table 2**.

**Table 2:** Mean levels of positive and negative mood before and after the psychosocial stressor (TSST) in endurance-trained men, resistance-trained men, and untrained men.

	Endurance (*n* = 12)	Resistance (*n* = 10)	Untrained (*n* = 12)
Positive mood			
Before TSST	2.27 ± 0.12	2.20 ± 0.19	1.95 ± 0.15
After TSST	2.17 ± 0.13	2.01 ± 0.17	1.70 ± 0.20
Negative mood			
Before TSST	0.27 ± 0.08	0.26 ± 0.09	0.23 ± 0.07
After TSST	0.73 ± 0.23	0.76 ± 0.24	0.99 ± 0.28

*Data are presented as mean ± SEM; the scale ranges from not at all (0) to extremely (4).*

## Discussion

This study tested whether individuals who engage regularly and long-term in either endurance or resistance training differ from untrained individuals in adrenal, cardiovascular, and psychological responses to a standardized psychosocial stressor. We found that the psychosocial stressor (TSST) significantly increased cortisol and heart rate responses, and worsened negative mood in all three study groups. Only the pattern of heart rate responses differed among the study groups, with untrained men showing higher cardiovascular stress responsiveness than trained men. Furthermore, the untrained group had significantly higher heart rate levels during the test than both the endurance and the resistance groups, whereas the two latter groups did not differ significantly from each other. We did not observe any significant group differences in cortisol and mood responses to the stressor.

Prior research has demonstrated that long-term endurance training is linked to lower cardiovascular responsiveness to laboratory stressors (e.g., Rimmele et al., 2007, 2009; Klaperski et al., 2013). Our data extend prior findings by showing that long-term resistance training may be associated with the same cardiovascular benefits. Both endurance-trained and resistance-trained men had lower heart rate levels during the stress protocol than untrained men, which may indicate overall higher cardiac performance of the trained groups. In addition, contrast analysis revealed that trained men (both endurance and resistance altogether) exhibited significantly lower heart rate responses to psychosocial stress compared to untrained men. The interpretation of that finding is limited by the observation that using the more conservative Bonferroni adjusted pairwise comparison between endurance-trained men and untrained men, and between resistance-trained men and untrained men were no longer significant. As pointed out in the results’ section, this, however, is by some considered a too conservative approach, favoring the null hypothesis (Perneger, 1998). Nonetheless, together our data provide some support for the protective role of regular physical training (both endurance and resistance) against stress.

The reported differences between trained men and untrained men cannot be attributed to different levels of chronic stress, or baseline HPA axis regulation. The study groups differed neither in the CAR nor in self-reported levels of perceived life stress. It is therefore unlikely that the lower responses of trained men to the induced, acute stressor were due to less stressful lives in general, or a changed baseline HPA axis regulation, when compared with untrained men. In contrast, it could be envisioned that trained men were more adapted to acute stressors. The cross-stressor adaptation hypothesis (Hamer et al., 2006; Sothmann, 2006) states that regular exercise leads to biological adaptations which result in a reduced reactivity of the sympathetic nervous system and the HPA axis to stressors in general. This hypothesis has been confirmed with experimentally induced stressors in a number of studies (see Klaperski et al., 2014, for the most recent overview). Thus, physical exercise may contribute to reduced physiological reactions not only to exercise-related stressors but also to psychosocial stressors (see also Sothmann, 2006; Gerber, 2008).

It is important to note that our data are partly in line with the assumption of the cross-stressor adaptation hypothesis with respect to cardiovascular reactivity, but to a lesser degree with respect to endocrine stress reactivity. Trained men showed significantly lower heart rate responses than the untrained group. For salivary cortisol, however, the effects were non-significant. Although both the endurance and the resistance groups showed lower mean absolute increase in response to stress than the untrained group, the group effect fell short of significance. The mechanisms underlying this dissociation are unknown. Our data merely mirror previous findings of relatively consistent effects of regular physical activity on cardiovascular responses after stress exposure (Forcier et al., 2006; Jackson and Dishman, 2006) but mixed results for endocrine stress responses (van Doornen and de Geus, 1993; Rimmele et al., 2009). Rimmele et al. (2009) suggest that the sympathetic nervous system (as the main regulator of heart rate) is more sensitive to the adaptive consequences of physical exercise than the HPA axis (as the main regulator of cortisol). The current findings would provide support for this differential effect as well. As we were studying long-term effects of training on autonomic and cardiovascular reactivity to stress, it is possible that initial reactivity differences among the groups disappear over time for the HPA axis, but not for the autonomic nervous system. To explore this possible explanation further would require to test for an association between training duration and reactivity in the endurance and resistance groups. Unfortunately, this test was not possible as we did not record the exact training duration of our participants other than the requirement that training had to be ongoing for at least 1 year. Thus, future studies should test for this possibility specifically.

With regard to the psychological stress responses, negative mood was intensified significantly in response to stress induction in all three groups. However, there were no differences observed among the groups. This is at odds with previous findings by Rimmele et al. (2007) who reported larger mood worsening in untrained individuals compared to trained individuals. On the other hand, Rimmele et al. (2009) found no group differences in negative mood worsening with regard to the level of physical activity, and Klaperski et al. (2013) reported even higher levels of negative mood after stress exposure in physically active individuals compared to inactive ones. So far, there is no existing explanation for this inconsistent evidence. Prior research demonstrated that physiological and psychological stress responses are not automatically linked to each other. As reported by Campbell and Ehlert (2012), significant correlations between self-reported emotional stress variables and physiological responses measured by heart rate and saliva cortisol emerged in less than 30% of 49 analyzed TSST studies. Future research is thus needed to find out moderating variables responsible for the dissociation between physiological and psychological stress responses.

We also controlled for action orientation in this study because action orientation might be related to both exercise level (Beckmann and Kazén, 1994) and stress responsiveness (Kuhl, 2001). In line with prior research (Beckmann and Kazén, 1994), physically trained men were more action-oriented than untrained men, but only with regard to the demand-related form of action orientation. Demand-related action orientation reflects personality differences in self-motivation (Kuhl, 1994b; Gröpel et al., 2014), which is of particular importance for regular exercise (Kendzierski, 1990). Kendzierski found that individuals high in demand-related action orientation exercised more regularly than those low in action orientation, as they were more able to overcome initial unwillingness, increases in demands, and tiredness. However, demand-related action orientation in the present study did not correlate with any measure of stress responsiveness. Thus, higher demand-related action orientation may be related to more regular exercise training but does not seem to moderate exercise-related benefits.

Some limitations deserve mention. As we used a cross-sectional study design, no causal conclusions can be drawn. Prospective longitudinal studies are necessary in this regard. Despite the specific prerequisites which qualified either for the endurance or the resistance group, it is unlikely that the endurance group never engaged in resistance training and the resistance group never engaged in endurance training. Every ambitious athlete needs to train both endurance and resistance to a certain extent. Thus, to separately test the effects of endurance and resistance exercise, long-term experimental studies with manipulations of the type of exercise are needed. Further, with 10–12 subjects per group, we might be on the lower side of detecting an effect. Finally, the generalizability of our results is limited to healthy young men. Replications with other samples would provide more insight into the influence of gender, age, and clinical factors on exercise-related stress adaptations.

## Conclusion

We examined stress responsiveness of individuals who participated regularly in either a long-term endurance or resistance training. Our results partly support the importance of regular exercise regardless of the type of exercise. Specifically, the data suggest that both endurance and resistance exercises, when performed over a longer period of time, may increase cardiac performance and reduce cardiovascular reactivity to acute psychosocial stress. Thus, regular physical activity seems to have health-promoting effects, regardless of whether it includes endurance exercise such as jogging, walking or biking, or resistance exercise such as lifting weights.

## Author Contributions

MU, JP, and MQ conceived and designed the study and revised the paper. MU performed the study. PG, MU, and MQ analyzed and interpreted the data. PG wrote the paper. PG, MU, JP, and MQ gave final approval of the manuscript to be published.

## Conflict of Interest Statement

The authors declare that the research was conducted in the absence of any commercial or financial relationships that could be construed as a potential conflict of interest.
